# Effects of Sodium Hexametaphosphate and Fluoride on the pH and Inorganic Components of *Streptococcus mutans* and *Candida* *albicans* Biofilm after Sucrose Exposure

**DOI:** 10.3390/antibiotics11081044

**Published:** 2022-08-03

**Authors:** Thayse Yumi Hosida, Juliano Pelim Pessan, Thamires Priscila Cavazana, Caio Sampaio, Leonardo Antônio de Morais, Douglas Roberto Monteiro, Alberto Carlos Botazzo Delbem

**Affiliations:** 1 Department of Preventive and Restorative Dentistry, School of Dentistry, São Paulo State University (UNESP), Araçatuba 16015-050, São Paulo, Brazil; thayse.hosida@unesp.br (T.Y.H.); juliano.pessan@unesp.br (J.P.P.); thamirescavazana@gmail.com (T.P.C.); caio.sampaio@unesp.br (C.S.); leonardo.a.morais@unesp.br (L.A.d.M.); douglas@unoeste.br (D.R.M.); 2 Postgraduate Program in Health Sciences, University of Western São Paulo (UNOESTE), Presidente Prudente 19050-920, São Paulo, Brazil

**Keywords:** biofilms, *Streptococcus mutans*, *Candida albicans*, fluorides, phosphates, hexametaphosphate, HMP

## Abstract

In order to improve the anticaries effects of fluoridated products, the supplementation of these products has been considered a promising alternative for caries control. This study evaluated the effects of sodium hexametaphosphate (HMP) and/or fluoride (F) on the inorganic components and pH of *Streptococcus mutans* and *Candida albicans* dual-species biofilms. The biofilms were treated 72, 78, and 96 h after the beginning of their formation with 0.25, 0.5, or 1% HMP-containing solutions with or without F (500 ppm, as sodium fluoride). F-containing solutions (500 ppm and 1100 ppm) and artificial saliva were used as controls. The biofilms were exposed to a 20% sucrose solution after the third treatment. Along with the biofilm pH, the concentrations of F, calcium, phosphorus (P), and HMP were determined. HMP, combined with F, increased F levels and decreased P levels in the biofilm fluid compared to that of the solution with 500 ppm F. Exposure to sucrose decreased the concentrations of all ions in the biomass, except for HMP; 1% HMP, combined with F, promoted the highest pH. It can be concluded that HMP affected the inorganic composition of the biofilm and exerted a buffering effect on the biofilm pH.

## 1. Introduction

Dental caries is a multifactorial, sugar biofilm-dependent disease [1] caused by acid-producing bacteria and fermentable carbohydrates that progressively results in enamel demineralisation [2]. *Streptococcus mutans* is one of the common etiological agents of dental caries because of its ability to colonise dental surfaces, metabolise carbohydrates, produce lactic acid, and thrive in acidic medium [3]. The formation of biofilms increases lactic acid production, primarily under exposure to sucrose, resulting in a reduced pH [3]. Although *S. mutans* is considered the major pathogenic agent related to dental caries [4], *Candida albicans* has often been associated with the cariogenic biofilm, especially in early childhood caries [5,6]. *C. albicans* is also one of the most frequently found fungi in human mucosa. It is usually present in polymicrobial biofilms on soft tissues and dental surfaces, and acts on microbial adherence and the progression of dentine cavitation; it also affects the microbial pathogenicity of microorganisms [7,8].

Sodium hexametaphosphate (HMP; [NaPO_3_]_6_) consists of an inorganic cyclophosphate commonly used as a commercial antimicrobial agent due to its property of increasing the permeability of the microbial wall [9] and dispersing the microbial biofilm [10]. Recent data have demonstrated that fluoridated dentifrices supplemented with HMP exert superior effects on enamel demineralisation and remineralisation compared with that of their counterparts without HMP [11]. Moreover, it was demonstrated that the addition of HMP has been shown to promote important changes in the biofilms formed in situ with regard to ionic saturation and extracellular polysaccharides production [11]. Furthermore, a recent in vitro study using a dual-species biofilm model (*S. mutans* and *C. albicans*) showed that the combined treatments of HMP with fluoride (F) markedly reduced *S. mutans* colony-forming unit (CFU) counts, metabolic activity, biomass production, and the extracellular matrix [12].

Despite the favourable results described above, the mechanism by which HMP acts on the cariogenic biofilm remains unclear, with scarce data on the effects of this cyclophosphate (alone or combined with F) on the biofilm pH or inorganic composition. Although the information on the effects of HMP on microorganisms is not completely clear, it is known that this phosphate has a strong affinity to cations such as Mg^2+^ and Ca^2+^, which results in the formation of ionic complexes in the bacterial cell walls, consequently affecting cell permeability. This property (i.e., chelating ability) confers antimicrobial activity to HMP, affecting parameters related to microbial viability and metabolism, which includes pH [13,14]. The concentrations of F, phosphorus (P), and calcium (Ca) in the biofilm (biomass and biofilm fluid) play important roles in the dental demineralization and remineralisation process [15]. Furthermore, the concentrations of these ions in the biofilms present an inverse relationship with the incidence of dental caries [16], possibly due to ion release from the biomass to biofilm fluid, thus favouring enamel remineralization [17]. Considering the aforementioned information, this study aimed to evaluate the effects of HMP, combined or not with F, on the pH and on Ca, P, and F concentrations in the biomass and fluid of dual-species biofilms of *S. mutans* and *C. albicans* prior to and after exposure to sucrose in vitro.

## 2. Results

### 2.1. Evaluation of the Number of Cells Prior to the Treatment of Biofilms

Mean (SD) CFU values prior to the treatments of the biofilms were 6.88 (0.20) Log_10_ CFU/cm^2^ and 6.78 (0.10) Log_10_ CFU/cm^2^, for *S. mutans* and *C. albicans*, respectively.

### 2.2. F, Ca, and P Levels in the Biofilm Fluid

**Figure 1:**
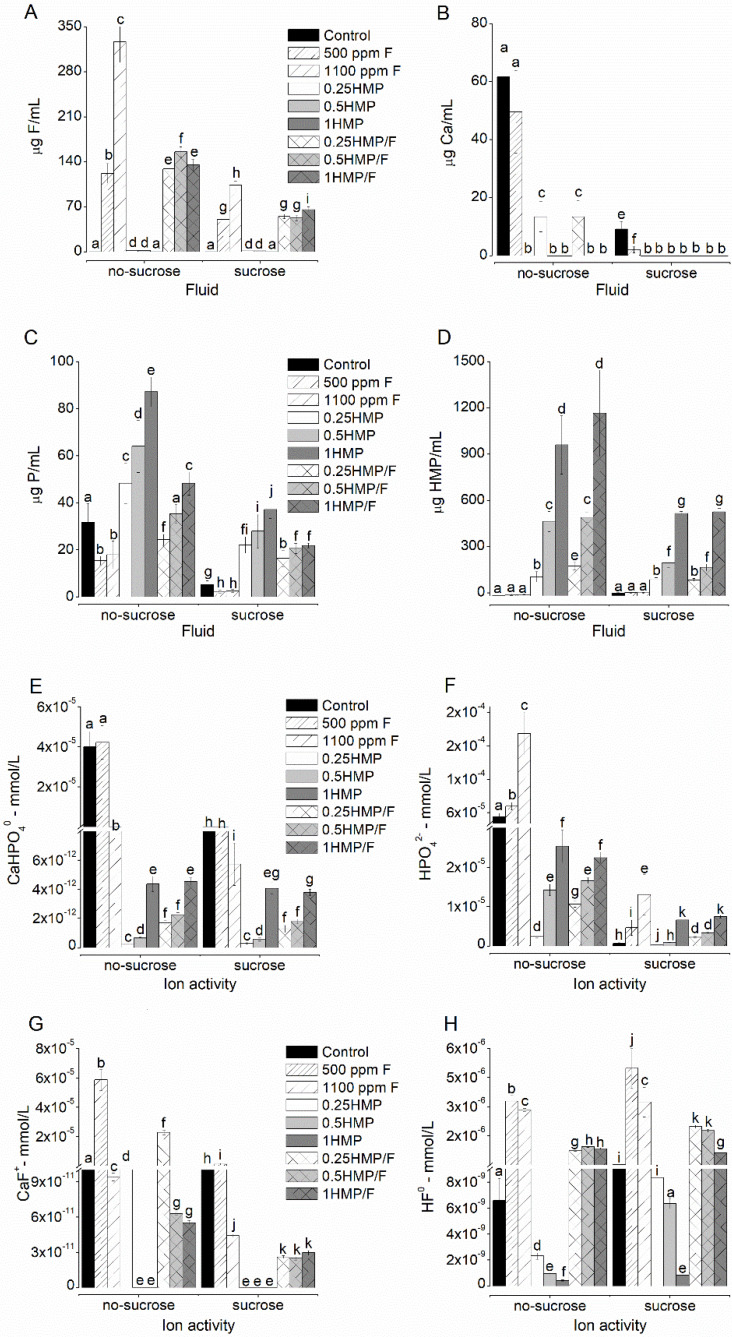
Mean values of F (**A**), Ca (**B**), P (**C**), and HMP (**D**), and ion activity of species CaHPO_4_^0^ (**E**), HPO_4_^−2^ (**F**), CaF^+^ (**G**), HF^0^ (**H**) in the biofilm fluid, before and after exposure to sucrose. The order of the groups on the x-axis follows the legend sequence, with the control group presented in the solid black color. The letters indicate comparisons among all values in each graph. Values with distinct letters indicate that there is a statistical difference between them, and values with equal letters indicate that there are no statistical differences between them. Bars indicate standard deviation (two-way ANOVA and Fisher’s LSD test, *p* < 0.05). Each assay was run in triplicate in three different experiments (*n* = 9).

F, Ca, and P ions and HMP in the biofilm fluid significantly decreased after exposure to sucrose (*p* < 0.001), regardless of the treatment group (Figure 1). The combined treatment of HMP with F led to higher fluoride levels when compared to that of 500 ppm F, whereas lower than 1100 ppm F for biofilms that were not exposed to sucrose (*p* < 0.001; Figure 1A). The groups treated with 1100 ppm F or with HMP at 0.5% and 1% (alone or together with F) did not show detectable levels of Ca in the biofilm fluid, regardless of the exposure to sucrose (Figure 1B). P levels were significantly higher in the groups exposed to HMP without F than in those treated with F, both prior to and after exposure to sucrose (*p* < 0.001; Figure 1C). A dose-response relationship was observed between the HMP concentrations in the biofilm fluid and treatment solutions (*p* < 0.001; Figure 1D).

### 2.3. F, Ca, and P Levels in the Biofilm Biomass

**Figure 2:**
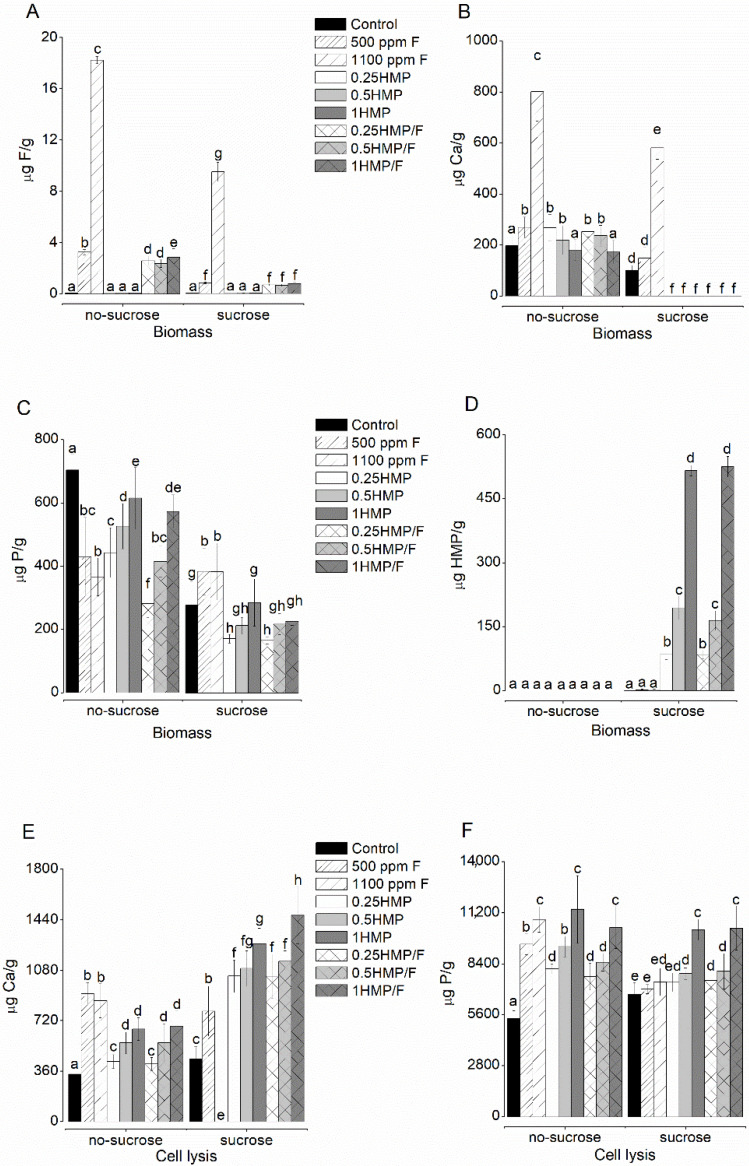
Mean values of F (**A**), Ca (**B**), P (**C**), and HMP (**D**) in the biofilm biomass, and Ca (**E**) and P (**F**) after cell lysis, before and after exposure to sucrose. The order of the groups on the x-axis follows the legend sequence, with the control group (black color) as the basis of it. The letters indicate the comparison among all values in each graph. Values with distinct letters indicate that there is a statistical difference between them, and values with equal letters indicate that there are no statistical differences between them. Bars indicate standard deviation (two-way ANOVA and Fisher’s LSD test, *p* < 0.05). Each assay was run in triplicate in three different experiments (*n* = 9).

Groups treated with HMP and F had significantly lower F values than those treated with 500 ppm F (*p* < 0.001), prior to exposure to sucrose (Figure 2A). After the cariogenic challenge (with a 20% sucrose solution for 3 min), F levels were significantly reduced in all tested groups, with no significant difference among biofilms treated with solutions containing 500 ppm F. The Ca ion was not detected in any of the groups treated with HMP after sucrose exposure (*p* > 0.069; Figure 2B). Biofilms treated with 1100 ppm F presented higher Ca concentrations than the other groups, both before and after exposure to sucrose (*p* < 0.001; Figure 2B). As for P levels (Figure 2C), the higher the HMP concentration in the treatment solution, the higher the *p* values in biomass before sucrose exposure; however, such a dose-response relationship was not significant after sucrose exposure. Furthermore, the cariogenic challenge reduced P levels in all groups, except for 500 ppm F and 1100 ppm F. Finally, HMP phosphate was not detected in biofilms of any HMP-treated group prior to sucrose exposure. Nonetheless, after exposure, a dose-response relationship between HMP levels in the biomass and the treatment solutions was observed (Figure 2D), with no significant differences between the counterparts with or without F.

After cell lysis, groups treated with HMP (with or without F), after sucrose exposure, had significantly higher Ca levels than those that were not exposed (Figure 2E). As for *p* values, all HMP-treated groups presented higher *p* values compared to the control before sucrose exposure, in addition to presenting a dose-response relationship according to their concentrations. The groups 0.5HMP, 1HMP, 0.25HMP/F, 0.5HMP/F, and 1HMP/F exposed to sucrose had significantly higher P levels than the control group (Figure 2F).

### 2.4. Biofilm pH

The pH of all biofilms decreased significantly after exposure to sucrose (*p* < 0.001). A dose-response relationship was observed between the F concentrations in the treatment solutions and the biofilm pH, both prior to and after the sucrose challenge. Treatment with HMP/F led to higher pH values compared to all other groups that were not exposed to sucrose (Table 1). Furthermore, the pH of biofilms treated with 0.25HMP/F and 0.5HMP/F was not significantly different from that observed for the 1100 ppm F group after exposure to sucrose. In addition, treatment with 1HMP/F led to the highest pH value in comparison to that of all other groups after sucrose exposure.

### 2.5. Determination of Ionic Activities and Degree of Saturation from the Biofilm Fluid

The biofilm fluid was supersaturated in relation to HA in the groups, following the order 0.25HMP/F > 500 ppm F = 0.25HMP (log values > 0); biofilm fluid was undersaturated for the other groups (log values < 0) in a medium without sucrose exposure (Table 1). When exposed to sucrose, the biofilm fluid of all groups was undersaturated with respect to HA. Only the groups 0.25HMP/F = 500 ppm F > control had biofilm fluid supersaturated with respect to CaF_2_, without sucrose exposure. When exposed to sucrose, only the 500 ppm F group was saturated in relation to CaF_2_, the other groups were undersaturated with higher or lower negative values (Table 1).

The estimated formation of CaHPO_4_^0^ (Figure 1F) was higher in the groups treated with 0.25HMP and 0.25HMP/F prior to exposure to sucrose, without a significant difference between these groups after sucrose exposure. A tendency for a dose-response relationship was observed as a function of HMP concentration, in combination or not with F. Regarding the formation of HPO_4_^2-^ (Figure 1H), a clear dose-response was observed as a function of HMP concentration, regardless of the presence of fluoride, before or after sucrose exposure. HPO_4_^2-^ formation was significantly reduced after sucrose exposure. The group treated with 1100 ppm F showed reduced CaHPO_4_^0^ formation and increased HPO_4_^2-^ formation in comparison to that of the group treated with 500 ppm F. The highest CaF^+^ formation was observed in the group treated with 500 ppm F. The combined treatment of F with 0.25HMP resulted in higher CaF^+^ formation than its counterpart without F (Figure 1E). The calculated formation of HF^0^ (Figure 1G) was higher in the groups combined with F both before and after sucrose exposure.

## 3. Discussion

This study assessed the effects of HMP and F, in combination or not, on the inorganic components and pH of a dual-species biofilm of *S. mutans* and *C. albicans*, prior to and after exposure to sucrose. HMP was shown to reduce the Ca concentration in the biofilm in a dose-dependent manner. Furthermore, treatment with HMP and F led to significant increases in F and P concentrations in the biofilm fluid. The pH was also influenced by treatments with HMP and/or F, which were higher when both compounds were in combination.

The production of lactic acid by *S. mutans* after sucrose exposure resulted in a reduced pH, which can be responsible for the progression of carious lesions under clinical conditions [2,3]. The treatment with HMP solutions (with or without F) had a great influence on biofilm pH, with 1HMP/F leading to higher pH values, both before and after sucrose exposure, in line with previously reported studies [18,19]. Regarding the influence of HMP alone, its effects on biofilm (both solid and fluid phases) seem to be associated with the buffering capacity of this cyclophosphate [20]. Thus, the presence of HMP in the fluid would promote H^+^ binding to OH^-^ on its structure, thus leading to an increased pH, especially for the highest concentration tested. Moreover, HMP can undergo hydrolysis and increase the availability of PO_4_^2-^ in the fluid phase, thus favouring the formation of large amounts of HPO_4_^2-^. Although previous data have suggested a strong buffering action of HMP using protocols focusing on de- and re-mineralisation [11], the effects on biofilms described in the present study can have an important impact on reducing acid diffusion into the enamel.

Regarding the combination of HMP with F, it is known that F decreases the acidogenicity of *S. mutans*, inhibits the synthesis of extracellular polysaccharides, and reduces gene expression associated with glycosyltransferases and glycolysis, leading to a pH drop in the biofilm [19]. It is known that F enters the bacterial cell wall by the reaction H^+^ + F^–^ ⇌ HF, which happens especially under acidic conditions (p*K*_a_ = 3.15), and crosses the cell due to a higher permeability of HF to bacterial cell membranes [21]. From a dose-response standpoint, the higher availability of F promoted by 1100 ppm F compared with that of 500 ppm F may have contributed to the maintenance of more neutral pH values considering the above-mentioned mechanism. Furthermore, the maintenance of a pH closer to neutral values in this group (1100 ppm F) may be associated with HPO_4_^2-^ formation, which also has a buffering effect [13].

The present data showed that HMP is present in the fluid phase of the biofilm as a function of its concentration in the solutions, and could be adsorbed on enamel after a cariogenic challenge. This allows the formation of an HMP layer on the enamel surface that is capable of adsorbing Ca, phosphate, and F. In the oral environment, this layer can retain charged ions and ionic species (such as Ca^++^ and CaF^+^) by replacing Na^+^ in its cyclic structure, leading to a reticular formation in which Ca^++^ bridges molecules of HMP [9]. In this sense, Ca^++^ available in the medium (from saliva) binds to HMP, plummeting Ca^++^ concentrations in the biofilm fluid, and consequently influencing its saturation with respect to HA and CaF_2_. Lower levels of Ca^++^ also explain the lower degree of saturation of the biofilm fluid related to CaF_2_ and the lower formation of CaHPO_4_^0^ and CaF^+^ [9]. F, Ca, and P concentrations in the biofilm and biofilm fluid decreased after sucrose exposure, in line with previous observations using different biofilm models [22]. Although such decreases have also been noted in groups treated with HMP-containing solutions, the combined treatment of 1% HMP and 500 ppm F was shown to be effective in maintaining neutral biofilm pH, even after sucrose exposure. In fact, biofilms treated with HMP-containing solutions (combined or not with F) led to a lower degree of saturation in relation to HA and CaF_2_ in comparison to the control and 500 ppm F, which could negatively affect the dynamics of demineralisation and enamel remineralization [21]. Notably, the biofilms in the present study were not formed on a mineralised substrate (e.g., enamel, dentin, or hydroxyapatite specimens), and did not mimic the salivary flow that occurs intraorally, both of which could provide conditions for ionic exchange (and consequently a source of Ca^++^), thus influencing the resulting degree of saturation.

Furthermore, it was demonstrated that Ca^++^ mediates F uptake by the biofilm in the form of precipitated minerals (CaF_2_) or by binding to the bacteria surface and/or the extracellular matrix of the biofilms [22]. In fact, the interaction of F and Ca by the use of different F-containing products has been demonstrated to promote significant beneficial modifications regarding variables related to enamel de-/re-mineralisation and interprismatic obliteration [23]. Regarding the HMP-containing solutions (with or without F), it was observed that the higher the HMP concentration in the solutions, the lower the Ca^++^ levels in the biomass, both prior to and after the exposure to sucrose. This trend may be related to the ability of HMP to chelate cations [24], resulting in HMP binding to Ca^++^ and, subsequently, to the bacterial cell wall (i.e., calcium bridging). This mechanism also seems to be responsible for the antibiofilm effects of solutions containing F and HMP on dual-species biofilms of *S. mutans* and *C. albicans* [12].

The ability of HMP to bind to metallic ions may be related to the increase in P levels in the biofilm as well, primarily in combination with F. It has been reported that yeasts are able to develop mechanisms for nutrient adsorption under deprivation conditions, as in the case of Mg^+^ sequestration [24], which is supposedly bound to HMP. These microorganisms may perform endocytosis and capture nutrients bound to the HMP molecule, later destroying it in the cytoplasm, and subsequently resulting in ion efflux back to the culture medium [24]. Thus, it is possible that HMP from the treatment solution promoted metal chelation in the culture medium, forming HMP–metal complexes that might have been captured by *C. albicans*.

Another aspect that deserves attention is the antimicrobial activity of HMP due to its ability to increase the permeability of the bacterial outer membrane and glucose transportation when it is bound to Mg^+^ present in the cell wall [9]. This binding justifies the absence of HMP in the biofilm. After sucrose exposure, acid production seemed to promote the release of phosphate from the cell wall, as observed in the present study. Notably, HMP concentrations of 0.5 and 1%, with or without F, promoted higher HMP and Ca levels bound to microorganisms, which are also bound to HMP molecules.

The availability of P in the biofilm fluid was directly related to the HMP concentrations (especially at 0.5 and 1%) in the treatment solutions, suggesting that this phosphate undergoes hydrolysis over time. This is a relevant result because Ca and P levels in the biofilm fluid directly influence the formation of CaHPO_4_^0^. It is believed that combined treatments of HMP with F promote the formation of CaF^+^ in saliva, which can react with HPO_4_^2-^, forming CaHPO_4_^0^ and HF^0^ [25]. The present results corroborate this hypothesis, given that HF^0^ is present in a larger proportion in the biofilm fluid of groups treated with HMP and F. It has been reported that CaHPO_4_^0^ neutral species are paramount for enamel remineralisation, as its diffusion coefficient into subsurface lesions is much higher than that of ionic calcium [25]. However, a limitation of the present study is that the only source of Ca^++^ was the culture medium (saliva), which limits ionic exchange, making it difficult to estimate CaHPO_4_^0^ formation. Nonetheless, the study protocol was intentionally planned to provide data on the effects of HMP only in the biofilm, without interference from other sources, such as hydroxyapatite or dental substrates, variables that could be included in future studies. 

## 4. Materials and Methods

### 4.1. Microorganisms and Growth Conditions

Strains from the American Type Culture Collection (ATCC) were included in the study. Cultures of *S. mutans* (ATCC 25175) and *C. albicans* (ATCC 10231) were seeded on brain heart infusion agar (BHI; Difco) and Sabouraud dextrose agar (SDA; Difco, Le Pont de Claix, France), respectively. *S. mutans* were incubated in 5% CO_2_ (at 37 °C), for 24 h, and *C. albicans* plates were incubated for 24 h at 37 °C. After growth on agar, *S. mutans* colonies were suspended in 10 mL of BHI broth (Difco) and statically incubated overnight in 5% CO_2_ at 37 °C. *C. albicans* was suspended in 10 mL of SDA broth (Difco) and incubated at 37 °C overnight with shaking at 120 rpm [26]. The cells were centrifuged at 8000 rpm, for 5 min, and washed twice with 10 mL NaCl (0.85%). The fungal cells were adjusted to 10^7^ cells/mL in artificial saliva (AS), using a Neubauer chamber. The number of bacterial cells were adjusted spectrophotometrically (640 nm) at a concentration of 10^8^ cells/mL in saline solution (0.85% NaCl) [27]. AS was prepared according to Lamfon et al. [28] and supplemented with sucrose [29], composed of: 2 g sucrose (Sigma-Aldrich, Burlington, MA, United States), 1 g yeast extract (Sigma-Aldrich, Burlington, MA, United States), 2.5 g bacteriological peptone (Sigma-Aldrich, Burlington, MA, United States), 0.5 g mucin type III (Sigma-Aldrich), 0.175 g NaCl (Sigma-Aldrich, Burlington, MA, United States), 0.1 g CaCl_2_ (Sigma-Aldrich, Burlington, MA, United States), and 0.1 g KCl (Sigma-Aldrich, Burlington, MA, United States), pH 6.8, in 500 mL deionized water. For the biofilm growth, a 4 mL suspension containing 1 × 10^7^ cells/mL *C. albicans* + 1 × 10^8^ cells/mL *S. mutans* in AS was added to 6-well microtiter plates (Costar-Coring, USA). These were then incubated at 37 °C for 72 h. Every 24 h, AS was refreshed at 50% (removal of 2 mL from the wells and addition of 2 mL of fresh AS). 

### 4.2. Evaluation of the Number of Cells Prior to the Treatment of Biofilms

To verify the formation of dual-species biofilms, the number of *S. mutans* and *C. albicans* cells prior to the treatment was assessed by counting CFUs. [12]. In brief, 0.85% NaCl was pipetted to the wells and the biofilms were scraped. The suspensions were serially diluted in 0.85% NaCl and plated on CHROMagar *Candida* (Difco) or in BHI agar supplemented with amphotericin B (7 μg/mL) (Sigma-Aldrich, Burlington, MA, United States) to count *C. albicans* and *S. mutans*, respectively. The plates were then incubated (24–48 h) at 37 °C.

### 4.3. Treatment of Biofilms and pH Measurement

The biofilms were treated 72, 78, and 96 h after the beginning of the biofilm formation, for 1 min [30,31]. The two first treatments were performed in order to achieve and verify the cumulative effect of the compounds, similarly to what happens in in vivo conditions after brushing with a fluoridated dentifrice. Regarding the last treatment, it was performed in order to evaluate the effect of the compounds on the biofilms right after the treatment [30,31].

The treatments were performed with HMP solutions of 0.25% (0.25HMP), 0.5% (0.5HMP), 1% (1HMP), 0.25HMP + 500 ppm F (0.25HMP/F), 0.5 HMP + 500 ppm F (0.5HMP/F), and 1HMP + 500 ppm F (1HMP/F), prepared by diluting the compounds in deionized water [12]. Solutions containing 500 and 1100 ppm F and pure AS (without F or HMP) were tested as controls. After the last treatment, the biofilms were gently washed with 1 mL AS (for 10 s) [12]. The biofilms were then scraped with a cell scraper and transferred, with the assistance of a pipette, to microtubes. For pH measurement, it was performed using a pH electrode (PHR-146 Micro Combination pH Electrode, Fisher Scientific, California, USA), previously calibrated with 7.0 and 4.0 pH standards [29]. The biofilm pH was measured in the total biofilm, before the separation of the biofilm fluid and biomass. Given that the biofilm pH should be measured immediately after the last treatment or the sucrose exposure (for those biofilms exposed to sucrose), the biofilm pH was determined before the separation of the solid and fluid phases.

In another set of experiments, after the last treatment, AS was removed from the wells, and the biofilms were submitted to a cariogenic challenge by their exposure to a 20% sucrose solution for 3 min [29]. The sucrose solution was then removed, and the biofilms were scraped from the wells and transferred to microtubes (within 1 min after removal of the sucrose solution). The pH analysis was performed as described above [29]. The sucrose challenge above was based on a previous study [29], which involved the evaluation of sucrose solutions at different concentrations administered at different duration periods in order to resemble clinical conditions. It was validated that a mixed biofilm of *S. mutans* and *C. albicans* exposed to a 20% sucrose solution for 3 min exhibited a pattern of pH change similar to that observed in vivo.

All the parameters analysed in this study (i.e., biofilm pH, and F, Ca, and P from the biofilm fluid and biomass) have been analysed in biofilms exposed or not to the sucrose challenge, in separate experiments.

### 4.4. Analysis of F, Ca, and P Levels in the Biofilm Fluid

The microtubes with scraped biofilms were then centrifuged (15,267× *g*) for 5 min (at 4 °C), and the biofilm fluid was collected [31]. F was determined by an ion-specific (Orion 9409 BN) and a reference electrode (Orion 900100), coupled to an analyser (Orion, Thermo Scientific, Beverly, USA). For calibration, F standards were prepared using known F concentrations. A total ionic strength adjustor buffer (TISAB II) was used under the same conditions as the samples at a 1:1 ratio [31]. Calcium was analysed by spectrophotometry (EONC Spectrophotometer, Biotek, USA) at 650 nm, according to the method described by Vogel, Chow, and Brown [32]. In summary, 5 μL of standards or samples, 50 μL of deionized water, and 50 μL of Arsenazo III were used.

Total phosphorus was determined as described by Fiske and Subbarow [33]. The analysis of P from HMPs was performed according to Anderson, Dingwall, and Stephen [34]. Briefly, 0.1 mL of sulfuric acid 5 M (Sigma-Aldrich) and 0.1 mL periodic acid (Sigma-Aldrich) were added to 0.1 mL samples. The samples were then placed in a boiling water bath (Tecnal, TE-054-MAG, Brazil) at 100 °C, for 1 h. After cooling, 0.4 mL deionized water, 0.2 mL 8% sodium sulfite (Sigma-Aldrich), and 0.1 mL 7% sodium molybdate (Sigma-Aldrich) were added to the samples. After being homogenized, 0.1 mL of 1% hydroquinone (Sigma-Aldrich) was added. After 30 min, the samples were read using a plate reader (EONC Spectrophotometer, Biotek) at 640 nm. For the determination of P from HMP in the samples exposed to sucrose, the boiling water bath process was performed at 60 °C for 6 h [31].

### 4.5. Analysis of F, Ca, and P Levels in the Biofilm Biomass

For the measurement of the inorganic components of the biofilm biomass, 0.5 mL HCl (0.5 mol/L) was pipetted to the microtubes containing 10.0 mg plaque wet weight and homogenised [35]. The resulting mixture was kept for 3 h (at room temperature), under constant stirring (at 120 rpm), and then centrifuged (11,000× *g*) for 1 min [36]. A volume of 0.4 mL of the liquid was removed and the same volume of NaOH (0.5 M) was added. F, Ca, and P were analysed as previously described for biofilm fluids [31,32,33,34].

### 4.6. Determination of HMP and Ca Levels after Cell Lysis

To quantify the HMP and Ca levels bound to the microorganisms, 50 µL of HCl (1 mol/L) was added to the microtubes containing 10 mg of biomass and homogenised. The microtubes were placed in boiling water (100 °C, 30 min) to promote cell lysis [37] and HMP hydrolysis [32], and were then centrifuged (11,000× *g*, 1 min) [37]. A volume of 0.4 mL of the liquid was removed and 0.4 mL NaOH (1 mol/L) was added for Ca [32] and P analysis [33].

### 4.7. Determination of Ionic Activities and Degree of Saturation from the Biofilm Fluid

The ionic activities (IA) of species related to enamel remineralisation (CaHPO_4_^0^, HPO_4_^2^, CaF^+^, and HF^0^) were determined from the Ca, F, and P levels (mmol/L) in the biofilm fluid [25]. In addition, the degree of saturation (DS) of the solid phases of hydroxyapatite (HA) and calcium fluoride (CaF_2_) was determined on a logarithmic scale (saturation index) using the PHREEQC Interactive (version 2.18.3, U.S. Geological Survey Branch of Information Services, Denver, CO, USA) speciation programme. All calculations were performed at 37 °C, a density of 1.0 g/cm^3^, and pH values in the biofilm as determined previously. The solution was undersaturated at log(DS) < 0, saturated at log(DS) = 0, and supersaturated at log(DS) > 0.

### 4.8. Statistical Analysis

Data were analysed using the statistical software SigmaPlot 12.0 (Systat Software Inc., San Jose, CA, USA). Data normality was verified by the Shapiro–Wilk test. Data were submitted to two-way analysis of variance, followed by Fisher’s LSD test, adopting a significance level of 5%. Each assay was run in triplicate in three different experiments (*n* = 9). The number of experiments was based on a previous study [31], considering a α-error of 5% and a β-error of 20%. This study followed the CRIS checklist for reporting in-vitro studies (Supplementary Material Table S1).

## 5. Conclusions

Based on the trends above, it can be concluded that: (1) HMP significantly affected the dual-species biofilms of *S. mutans* and *C. albicans*, increasing HMP, F, and P concentrations in biofilm fluid; and (2) HMP and F were able to maintain the biofilm pH neutral, even after exposure to sucrose. This study provides new information on the mechanism of action of HMP and helps to explain the combined effects of this cyclophosphate with F on enamel de- and re-mineralisation processes and oral biofilms.

## Figures and Tables

**Table 1:** Mean (SD) pH of the biofilm and degree of saturation in relation to hydroxyapatite (HA) and calcium fluoride (CaF_2_) in the biofilm fluid, before and after exposure to sucrose.

Groups	pH		* Degree of Saturation (log)			
			HA		CaF_2_	
	No Sucrose	Sucrose	No Sucrose	Sucrose	No Sucrose	Sucrose
Control	6.10 ^a,A^ (0.07)	4.77 ^a,B^ (0.02)	−3.44 ^a,A^ (0.10)	−13.18 ^a,B^ (1.04)	1.30 ^a,A^ (0.24)	−5.12 ^a,B^ (0.19)
500 ppm F	6.62 ^b,A^ (0.22)	6.20 ^b,B^ (0.32)	2.98 ^b,A^ (0.93)	−9.65 ^b,B^ (1.62)	2.85 ^b,A^ (0.13)	0.77 ^b,B^ (0.18)
1100 ppm F	7.08 ^c,A^ (0.16)	6.49 ^c,B^ (0.31)	−29.40 ^c,A^ (1.52)	−34.62 ^c,B^ (1.02)	−3.23 ^c,A^ (0.38)	−4.24 ^c,B^ (0.06)
0.25HMP	6.46 ^bd,A^ (0.26)	5.52 ^d,B^ (0.25)	2.14 ^b,A^ (0.74)	−39.46 ^d,B^ (0.86)	−4.61 ^d,A^ (0.21)	−11.36 ^d,B^ (0.03)
0.5HMP	6.60 ^bf,A^ (0.05)	5.73 ^e,B^ (0.14)	−29.80 ^c,A^ (0.52)	−36.26 ^e,B^ (0.43)	−11.22 ^e,A^ (0.06)	−11.02 ^e,B^ (0.26)
1HMP	6.87 ^cg,A^ (0.23)	6.51 ^c,B^ (0.10)	−28.77 ^c,A^ (1.12)	−31.25 ^f,B^ (0.70)	−11.31 ^e,A^ (0.20)	−11.37 ^d,A^ (0.05)
0.25HMP/F	7.22 ^e,A^ (0.08)	6.52 ^c,B^ (0.12)	5.23 ^d,A^ (0.92)	−31.84 ^f,B^ (0.87)	2.32 ^b,A^ (0.25)	−4.79 ^f,B^ (0.05)
0.5HMP/F	7.27 ^e,A^ (0.06)	6.58 ^c,B^ (0.18)	−27.46 ^e,A^ (0.54)	−31.31 ^f,B^ (0.66)	−4.04 ^f,A^ (0.04)	−4.83 ^f,B^ (0.08)
1HMP/F	7.28 ^e,A^ (0.10)	6.99 ^f,B^ (0.13)	−27.90 ^e,A^ (0.44)	−29.29 ^g,B^ (0.43)	−4.20 ^f,A^ (0.11)	−4.68 ^f,B^ (0.06)

Distinct lower-case letters indicate statistical differences among the groups for each variable. Distinct upper-case letters indicate statistical differences between no sucrose and sucrose (two-way ANOVA and Fisher’s LSD test; *p* < 0.05). Each assay was run in triplicate in three different experiments (*n* = 9). * The degree of saturation was given on a logarithmic scale as the saturation index, log(DS). Negative or positive log values express the degree of saturation of the biofilm fluid: undersaturated < 0, saturated = 0, and supersaturated > 0.

## Data Availability

The data presented in this study are available on request from the corresponding author.

## Supplementary Materials

The following supporting information can be downloaded at: https://www.mdpi.com/article/10.3390/antibiotics11081044/s1, Table S1: CRIS checklist for reporting in-vitro studies based on previously guidelines *.
