# Supplementary material for: Effects of Sodium Hexametaphosphate and Fluoride on the pH and Inorganic Components of Streptococcus mutans and Candida albicans Biofilm after Sucrose Exposure
Source: Antibiotics (Basel). 2022 Aug 3;11(8):1044. doi: 10.3390/antibiotics11081044 (PMC9405115; doi:10.3390/antibiotics11081044)
Supplement: Supplementary file 1 [file antibiotics-11-01044-s001.zip › antibiotics-1811843-supplementary.pdf]

Supplementary Materials

Table S1. CRIS checklist for reporting in-vitro studies based on previously guidelines *.

Section/topic	Checklist item	
**Abstract**	Item 1. Structured summary of trial design, methods, results, and conclusions	Yes
**Introduction**		
Background and objectives	Item 2a. Scientific background and explanation of rationale	Yes
	Item 2b. Specific objectives and/or hypotheses	Yes
**Methods**		
Intervention	Item 3. The intervention for each group, including how and when it was administered, with sufficient detail to enable replication	Yes
Outcomes	Item 4. Completely defined, pre-specified primary and secondary measures of outcome, including how and when they were assessed	Yes
Sample size	Item 5. How sample size was determined	Yes
Randomization:		
Sequence generation	Item 6. Method used to generate the random allocation sequence	Not applicable
Allocation concealment mechanism	Item 7. Mechanism used to implement the random allocation sequence (for example, sequentially numbered containers), describing any steps taken to conceal the sequence until intervention was assigned	Not applicable
Implementation	Item 8. Who generated the random allocation sequence, who enrolled teeth, and who assigned teeth to intervention	Not applicable
Blinding	Item 9. If done, who was blinded after assignment to intervention (for example, care providers, those assessing outcomes), and how	Not applicable
Statistical methods	Item 10. Statistical methods used to compare groups for primary and secondary outcomes	Yes
**Results**		
Outcomes and estimation	Item 11. For each primary and secondary outcome, results for each group, and the estimated size of the effect and its precision (for example 95% confidence interval)	Yes
**Discussion**		
Limitations	Item 12. Trial limitations, addressing sources of potential bias, imprecision, and, if relevant, multiplicity of analyses	Yes
**Other information**		
Funding	Item 13. Sources of funding and other support (for example suppliers of drugs), role of funders	Yes
Protocol	Item 14. Where the full trial protocol can be accessed, if available	Yes

* https://doi.org/10.1016/j.jebdp.2012.10.001 (accessed on 23 July 2022). * https://pubmed.ncbi.nlm.nih.gov/25125839/ (accessed on 23 July 2022).
